# Environmental cleaning barriers and mitigation measures identified through two initiatives in four countries, 2018–2023: a commentary

**DOI:** 10.1186/s13756-024-01491-5

**Published:** 2024-11-08

**Authors:** Molly Patrick, Claire Kilpatrick, Julie Storr, Giorgia Gon, Tuan Huynh, Phung Manh Thang, Damilola Adeniyi, Folasade Ogunsola, Fatuma Manzi, Ir Por, Bernice Sarpong, Yovitha Sedekia, Ma Sokvy, Vouchnea Tang, Sreytouch Vong, Wendy Graham

**Affiliations:** 1 Division of Healthcare Quality Promotion, Centers for Disease Control and Prevention, Atlanta, GA USA; 2 KSHealthcare Consulting Ltd, Gateshead, UK; 3 London School of Hygiene and Tropical Medicine, London, UK; 4 University Medical Center, Ho Chi Minh City, Vietnam; 5 Cho Ray Hospital, Ho Chi Minh City, Vietnam; 6 Centre for Infection Control and Patient Safety (CICaPS) College of Medicine, University of Lagos, Lagos, Nigeria; 7 Ifakara Health Institute, Ifakara, Tanzania; 8 National Institute of Public Health, Phnom Penh, Cambodia; 9 WaterAid, Melbourne, Australia; 10 Mwanza Intervention Trials Unit, Mwanza, Tanzania; 11 WaterAid, Phnom Penh, Cambodia; 12 Independent Consultant, Phnom Penh, Cambodia

**Keywords:** Healthcare environment, Cleaning, Cleaners, Infection prevention, Training, Resources, Leadership, Investment, Adaptation, Professionalization

## Abstract

In recent years, there has been increased attention on the importance of healthcare environmental cleaning, including the need to professionalize and support the workforce responsible for performing cleaning. Global agendas and strategies on infection prevention and control (IPC) and water, sanitation and hygiene highlight the need for improvements to this sector, particularly in resource-limited healthcare facilities in low- and middle-income countries. Correspondingly, several resources have been developed that aim to (1) improve professional training of cleaners and (2) improve implementation of best practices in resource-limited settings. This commentary seeks to provide insight into the barriers and facilitators to implementing these resources, drawing on the practical experience from two initiatives across four countries from 2018 through 2023. Several common barriers were identified across the diverse settings, including (1) low empowerment and status of the workforce, (2) low pay, inadequate staff time for the high workload needed to achieve best practices and high turnover of staff, and (3) a lack of connection and integration of environmental cleaning with IPC and patient safety efforts at the participating hospitals. Despite barriers, local teams identified effective mitigation measures. While considerable time and effort will be needed to truly overcome these barriers, there are opportunities to build upon attention and momentum on this topic and IPC initiatives in resource-limited settings in low- and middle-income countries. We propose several broader actions, all of which require local leadership and context-specific approaches.

## Background

Environmental cleaning in healthcare facilities is a fundamental infection prevention and control (IPC) measure and therefore essential to patient and healthcare worker safety. The staff whose primary responsibility is to clean—hereafter referred to as “cleaners”—are thus contributing to important goals of preventing healthcare-associated infections (HAIs) and reducing antimicrobial resistance (AMR) in healthcare settings. These key staff are part of the second largest group among the estimated 65.1 million members of the global health workforce [1]. With such an important role and sizeable representation, it would be reasonable to expect that cleaners are valued and supported. However, across the globe, this is often not the case, with cleaners described as invisible in the workplace, unempowered, untrained, and unable to undertake their daily routines effectively and safely owing to inadequate infrastructure, equipment and supplies [2].

The neglect of environmental cleaning in healthcare has been raised recently through global agendas, including the World Health Assembly resolution on water, sanitation and hygiene (WASH) in 2019 [3] and the global strategy for IPC approved by all countries in 2022 [4], and through calls to action in scientific publications. Storr et al. [5], for example, noted five considerations to move the agenda forward: enhance the available data, implement norms and standards, combine advocacy efforts, revisit investment, and address the research gaps. In 2023, a research prioritization exercise was undertaken, which identified 12 major gaps in evidence for healthcare cleaning in resource-limited settings, including, for example, research into options for the professionalization of cleaners [6].

Recognition of the need to advance this sector globally has led to the creation of several resources for improving (1) professional training of cleaners and (2) implementation of best practices in low- and middle-income countries (LMICs) [7–10]. With the aim to contribute to future improvement efforts, here we present a synthesis of lessons on the barriers to implementing such environmental cleaning resources from two initiatives in four countries spanning the period 2018–2023 and the mitigation measures employed to overcome these barriers. Several broader actions are also proposed.

## Overview of resources and initiatives

### Initiative #1: TEACH CLEAN / World Health Organization (WHO) training package (Cambodia and Tanzania)

**Fig. 1:**
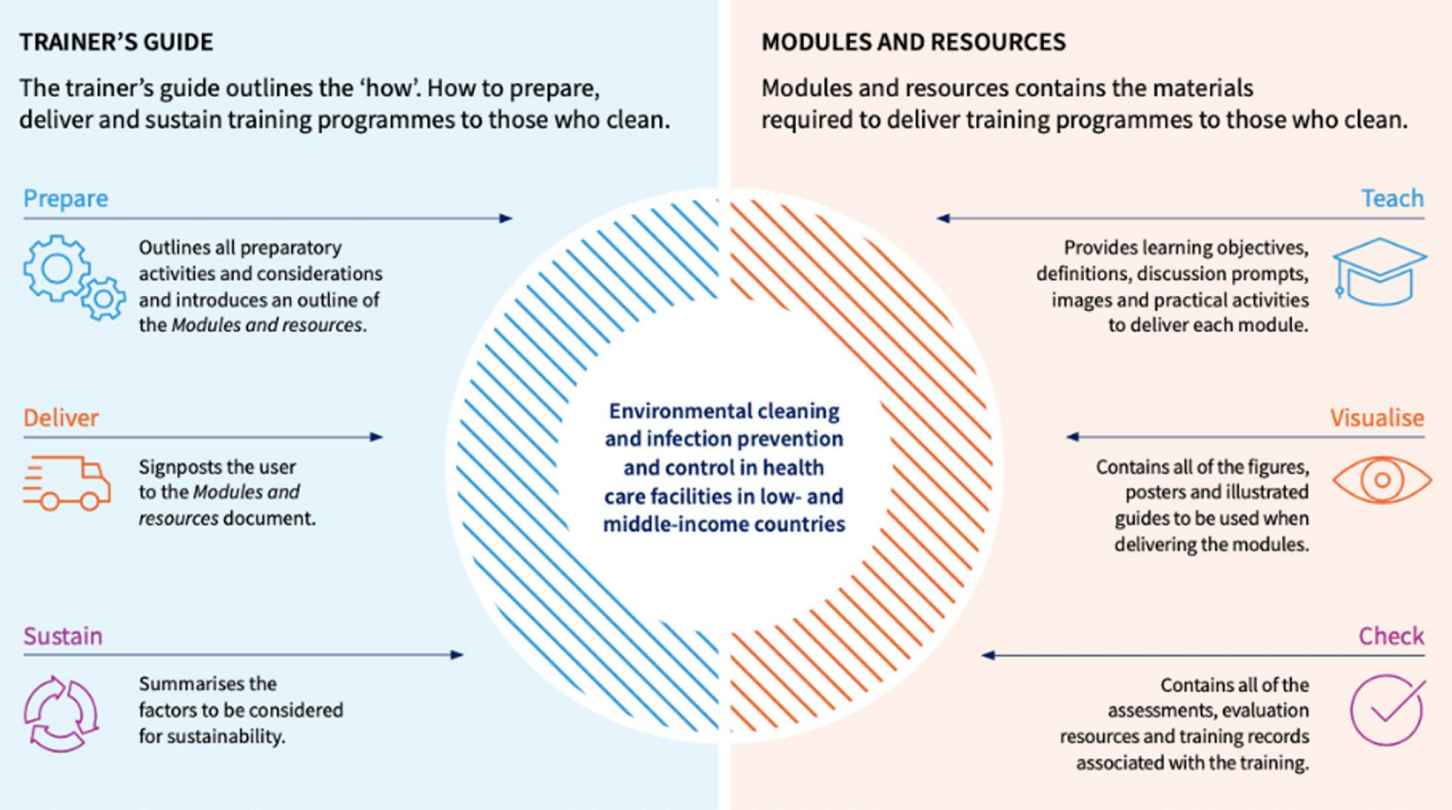
Outline of WHO two-part training package [7]

The TEACH CLEAN and WHO training packages are interrelated resources. These training packages are for training cleaners and are based on a training of trainer model. The TEACH CLEAN package was originally created by The Soapbox Collaborative and launched in 2018, and subsequently hosted by the London School of Hygiene and Tropical Medicine (LSHTM) [11]. The WHO built upon this, revising and updating the IPC content, and released an updated, two-part package in 2022 [7, 8] (see Fig. 1).

Over a five-year period, TEACH CLEAN was implemented and evaluated by The Soapbox Collaborative and LSHTM in collaboration with national and international organizations, including Ministries of Health and WaterAid, in four main countries: Cambodia, Gambia, Myanmar and Tanzania [12] and LSHTM [13]. Robust process and outcome evaluations were undertaken in three hospitals in Tanzania and 13 hospitals in Cambodia. This commentary draws specifically on the experiences from Tanzania and Cambodia: further details on the methods used in these evaluations can be found in WHO [14].

### Initiative #2: US Centers for Disease Control and Prevention (CDC) cleaning program implementation toolkit (Nigeria and Vietnam)

**Fig. 2:**
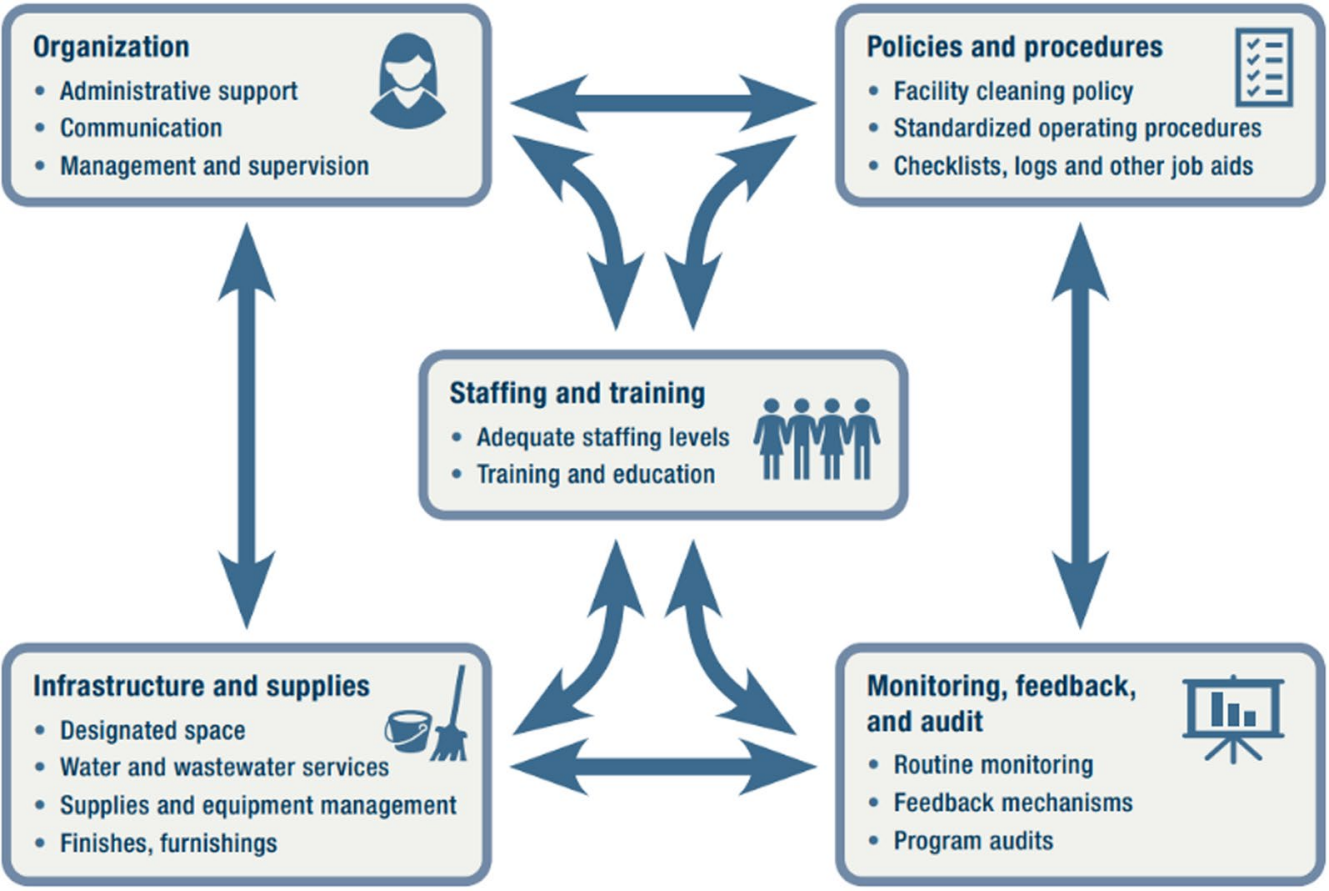
Environmental cleaning program elements targeted within the toolkit [9]

**Fig. 3:**
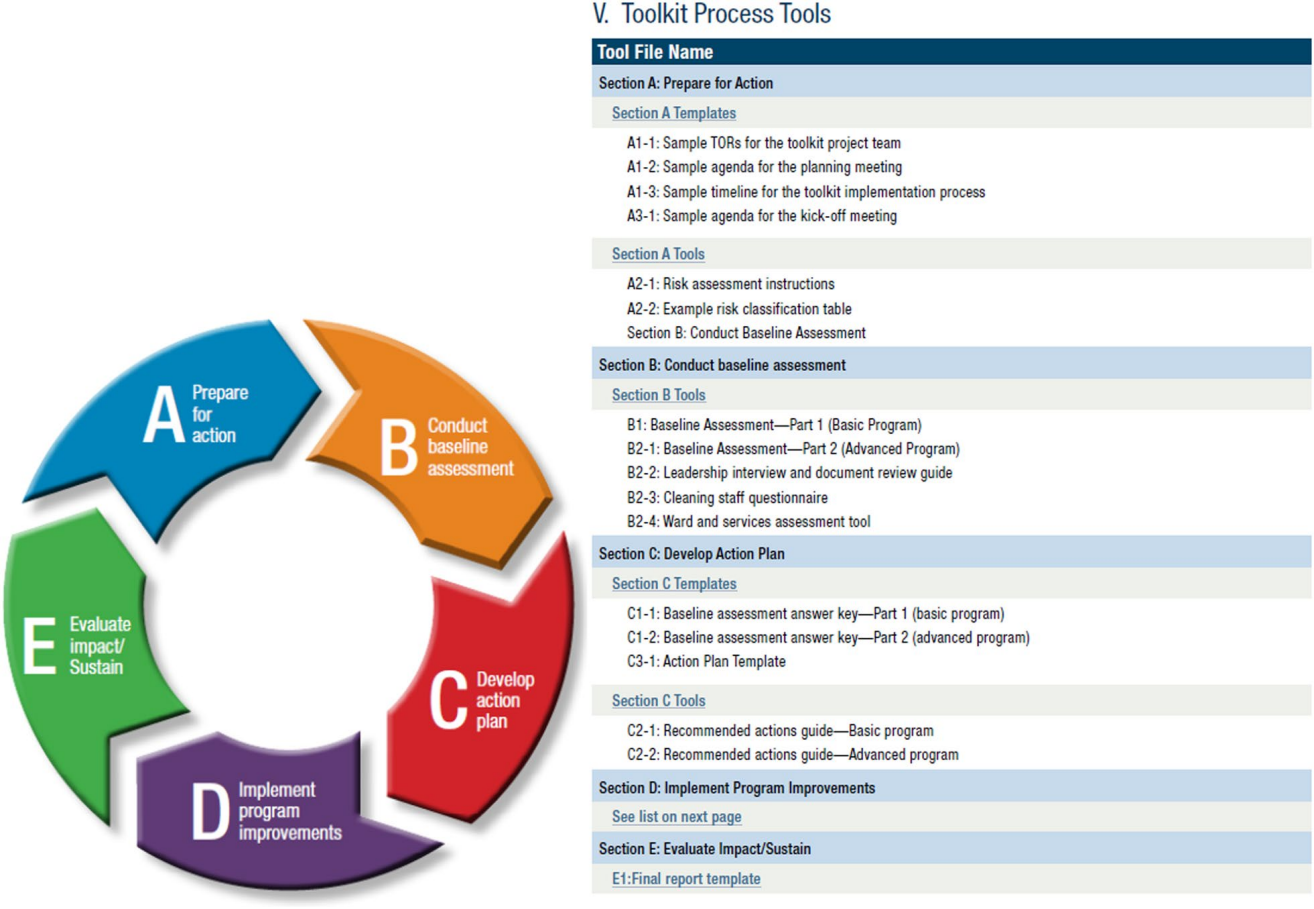
Summary of 5-step approach and tools within CDC Toolkit [9]

CDC’s Environmental Cleaning Program Improvement Toolkit [9] aims to support healthcare facilities with implementing the environmental cleaning program elements described within the CDC/Infection Control Africa Network (ICAN) Best Practices for Environmental Cleaning in Healthcare Facilities in Resource-Limited Settings, by providing a practical quality improvement approach and accompanying resources [10] (see Figs. 2 and 3).

During 2020–2021, this toolkit was piloted in Lagos, Nigeria (two neonatal wards in a tertiary care hospital) and Ho Chi Minh City, Vietnam (1–2 departments within each of two tertiary care hospitals). The goal of these pilots were to refine content and validate the approach prior to the Toolkit’s final publication and was conducted as part of ongoing quality improvement initiatives.

## Lessons identified

For initiative #1, lessons were synthesized from the process and outcome evaluation reports, papers, briefings and dissemination workshops in-country, and consultations with key individuals in the implementing agencies.

For initiative #2, lessons were synthesized via the completion of a case study form regarding the overall project experience by each hospital team. The form prompted them to describe (1) what was done and achieved, (2) what challenges were encountered and (3) what solutions were sought/implemented to address the challenges. Local partners in each country undertook additional informal interviews and discussions with the hospital staff to gain further insight into their responses and probe for more details on identified themes.

There were three main barriers to the implementation identified across the focus countries (Cambodia, Nigeria, Tanzania and Vietnam) and corresponding mitigation measures.

## Barrier 1: workforce profile

All projects were undertaken across a diverse range of settings, and all highlighted commonalities in the impact that the workforce profile presented to training initiatives. This centered around cleaners being primarily female, with a low level of education and literacy, and having a lack of status and empowerment within their roles. These characteristics posed challenges to training initiatives and necessitated considerable tailoring and adaptation of available training resources and the development of new modalities.

The materials from the TEACH CLEAN/WHO package were specifically developed to address these workforce profile challenges, including, for example, the use of mainly pictorial materials with little written content as training aides. However, the experiences across the settings outlined in this paper highlight that considerable time and resources are required to contextualize training content and approaches, even when these practical resources are already available.

### Mitigations: contextualization and innovation

In the settings where training initiatives were undertaken, the following activities helped to mitigate the challenges of workforce profile and so facilitate the training process: (1) translation of materials into local languages, the use of images from the local context, and incorporation of context-specific names for equipment or cleaning products and (2) an innovative facilitator in two of the settings was the creation of video resources to supplement written and pictorial content. In Cambodia, locally produced videos demonstrating specific cleaning techniques and procedures were developed for trainees. In Nigeria, an animated training video was developed which included narration in Pidgin English, given this was the primary oral language of most of the cleaning staff trainees. These efforts to deliver tailored and appropriate training programs contributed to cleaners feeling valued. Additionally, these trainings were the first professional development opportunity that most had been offered.

## Barrier 2: low pay, high workload and turnover

Low pay was also a common barrier across the settings, necessitating cleaners to take on second jobs and often contributing to staff turnover. These factors limited gains from training and brought to light an inability to perform daily cleaning according to best practices. In Cambodia and Tanzania, the low wages meant that cleaners often had other jobs to support themselves and this placed practical limits on the number of hours they were available each day. A consequence of the trainings was to apply further strain on cleaners, as it increased the expected number of tasks and workload; for example, manually cleaning and disinfecting reusable patient care equipment according to best practice was a new task for cleaners in Cambodia that required considerable additional time. In the hospitals in Cambodia, supervisors felt that it was difficult to place these extra demands on cleaners because they feared they would quit. In both settings, achieving daily cleaning of the patient zone was also a major challenge given the lack of full-time staff. In Tanzania, turnover after training was identified as a major challenge. Likewise in Nigeria, there were challenges relating to high turnover after the major training initiative had been conducted; however, in this setting, this was attributed both to low pay and expectations of long work hours, specifically 12-h shifts over 7-day periods with 2 weeks on and 1 week off.

### Mitigations: task shifting, prioritization and efficiency

Several measures were employed to help mitigate the barriers across the different countries: (1) in Tanzania and Cambodia, task shifting was attempted, whereby nurses and other auxiliary staff were trained on cleaning and supplemented efforts during certain day(s) of the week, and repeat as well as refresher training were conducted to ensure training of new cleaners; (2) in Cambodia, efforts also focused around how to prioritize cleaning schedules and tasks, such as increasing routine cleaning of high-touch surfaces; (3) to address turnover in Nigeria, the hospital developed more modular, on-the-unit training approaches as one way to reduce the time and resources required during the initial multi-day training activity.

## Barrier 3: lack of integration with IPC and clinical staff

A lack of oversight of environmental cleaning and cleaners by IPC staff at hospitals was noted, as well as a lack of relationship (e.g., coordination, collaboration) between clinical staff and cleaners at the unit/ward level. In Nigeria and Vietnam, lack of oversight (outsourced in both settings) was described as a barrier to making improvements. In Nigeria, the IPC team did not have input into the contract terms, which prevented any influence on the training required for cleaners as well as any ability to provide recommendations on the type and quality of cleaning products, supplies and equipment in use. In Vietnam, while overall there was more engagement by the hospitals’ IPC teams in the cleaning contract process (e.g., the hospital cleaning policy was used to inform the bidding process), there were gaps identified in the quality of routine monitoring which was led by the vendor with no role for the IPC team.

A disconnect of clinical staff from the environmental cleaning process and workforce was also identified. In Nigeria, Cambodia and Tanzania, some of the clinical staff participating in the project reported that, prior to the project, they had under-appreciated that environmental cleaning and cleaners played a role in patient safety within their departments. Lack of clarity on roles and responsibilities between cleaners and clinical staff and the impact on performance was also highlighted. At one hospital in Vietnam, while vendor-provided cleaners had been previously trained, the project team helped identify that the clinical staff on the unit with equipment cleaning responsibilities had not received any standard training on these duties. In the hospitals in Cambodia and Tanzania, there was a similar barrier encountered in terms of ambiguity around who cleans what, between clinical staff and cleaners.

### Mitigations: leadership engagement and inclusivity

To address the disconnect between IPC and environmental cleaning, an important facilitator was hospital leadership engagement. In Vietnam and Nigeria, leadership and administration supported IPC teams to either develop or update existing environmental cleaning policies and committed to aligning cleaning service contracts with these policies moving forward. In Vietnam, leadership also supported the IPC department to collaborate with the vendor to refine monitoring requirements and develop systems for sharing monitoring results between the vendor and the hospital IPC department. This new connection also allowed the IPC department to train the vendor on monitoring methods to improve the quality of the data collected. In Nigeria, leadership support of the project even extended to participation in the large training initiative, wherein the Chairman Medical Advisory Committee addressed trainees. Clinical staff, including nurses and unit leadership, also participated in the training in Nigeria, which helped improve awareness and engagement of these staff in environmental cleaning and the workforce. In Tanzania and Cambodia, leadership support was also critical, particularly in supporting clinical staff to take on the role of linking IPC and environmental cleaning—becoming so-called “champions”. These individuals were the first to be trained and then trained the cleaners at their hospitals. Crucially these champions also provided continuous supportive supervision with tools such as observation checklists, florescent gel monitoring and updated cleaning schedules. In Tanzania, the inclusion of nurses/midwives in the training at each hospital also helped to achieve a common understanding of cleaning standards and fostered better relations with the cleaning staff. Additionally, in Tanzania, for example, during feedback of results to Ministry of Health stakeholders, the importance of integration with the “five-star quality improvement system” was highlighted (Yahya & Mohamed, 2018).

## Implementation barriers relating to outsourcing

Environmental cleaning services were partially outsourced via contracts with third-party companies in Nigeria and Tanzania, and completely outsourced in Vietnam. In these projects, outsourcing was reported to further exacerbate the challenges related to workforce training.

First, in Nigeria, Tanzania and Vietnam (one of the hospitals), cleaners were provided by companies that either did not provide any training or did not provide training specific to healthcare cleaning. In Tanzania, most of the contracted staff had received no formal training. In Nigeria and Vietnam, while some of the contracted staff had received basic training on cleaning (and performed cleaning in other institutional settings), the training was not specific to healthcare cleaning techniques and best practices. Furthermore, there was a lack of appreciation at the company that environmental cleaning in a hospital was different from other settings, such as office or other commercial settings.

Secondly, the nature of the contract periods led to turnover on at least an annual basis. In Nigeria and Vietnam (both hospitals), the actual vendor (and all the staff) changed either directly before or during the implementation of the toolkit pilots, leading to a complete turnover of cleaning staff. Even in the one hospital in Vietnam where the vendor had experience in healthcare cleaning, there was still a need to support training specific to the hospital protocols, which created challenges and workload for IPC on an annual basis when contracts were re-advertised.

An additional insight from Tanzania was the complexity of supervision when cleaners were contracted, with essentially dual supervision from the company and the hospital clinical staff. There were also complexities when cleaning services were partially outsourced; in this case of mixed cleaning teams, there were also some reported issues with conflict or competition, given different pay rates and expectations.

## Conclusions and recommendations

Recent global initiatives targeted at environmental cleaning in healthcare settings in LMICs have supported the development of freely available resources for local adaptation and use. These resources provide some support for the needed empowerment and support for cleaners and the implementation of environmental cleaning best practices. However, these experiences from across diverse countries highlight that considerable barriers to progress persist, including that cleaners continue to be a neglected workforce and that cleaning exists as an “orphan” priority, with unresolved responsibility and accountability, within the healthcare system.

While some of the employed mitigations may be challenging to sustain due to level of required resources (e.g., repeated training in the face of frequent staff turnover), these projects identified several successful mitigations that are low resource or no resource interventions. For example, ensuring context-specific content (e.g., photographs) are used in training materials, as already suggested in global training resources, and developing task shifting approaches by engaging clinical staff in environmental cleaning efforts in the context of staffing shortages. Importantly, all the mitigation measures described herein required local experts to lead and champion efforts, as well as hospital leadership engagement and a culture of inclusivity. As outlined by Peters et al. [15] and Browne et al. [16], a multimodal approach is likely to be important in achieving long-term success with any cleaning improvement regardless of the resources or tools are applied.

Based on the lessons from these initiatives and building upon global efforts to advance environmental cleaning as a key IPC measure in LMICs, we propose several actions and considerations to address barriers to implementation:

- Use freely available training materials from trusted sources to support the development of a suite of national level, contextually specific, standardized training resources (including development of locally appropriate video resources) and make these available to both the public and private sectors. These actions are in line with recommendations within the Global IPC Strategy (Strategic direction 4: IPC knowledge of health and care workers and career pathways for IPC professionals) [4]
- Ensure national IPC and WASH experts, professional associations and governments lead contextualization efforts, ideally with national guidance, policy and implementation resources as key references. Such action supports engagement of health system decision-makers to prioritize the needed investment in environmental cleaning and provides further support for professionalization of the cadre.
- Improve understanding of the importance of integration and alignment of environmental cleaning activities within IPC and WASH initiatives in healthcare and ensure clear leadership for healthcare environmental cleaning at national level. While this leadership may differ depending on the governance structure in each country, it is essential to bring healthcare environmental cleaning under broader initiatives to improve patient safety and quality of care.
- Where appropriate, embed environment cleaning improvement within IPC initiatives at national and healthcare facility level, including training programs, performance-based financing, and quality improvement frameworks. Supportive supervision from IPC and clinical staff is also an essential ingredient to ensure environmental cleaning programs are sustained.
- Share both learning and outputs across countries to prevent wheel reinvention, for example, using existing global networks. This could support development of the minimum requirements for implementation of environmental cleaning programs at the health system level.

## Data Availability

Data sharing is not applicable to this article as no datasets were generated or analysed during the current study.

## Abbreviations

IPC: Infection prevention and control
AMR: Antimicrobial resistance
HAI: Healthcare-associated infection
WASH: Water, sanitation, and hygiene
LMIC: Low- and middle-income country
WHO: World Health Organization
CDC: Centers for Disease Control and Prevention
ICAN: Infection Control Africa Network
